# The First Year of Successful Execution & Examination of Integrated Modular Curriculum 2k23 by UHS Pakistan

**DOI:** 10.12669/pjms.40.5.9951

**Published:** 2024

I want to bring your attention to the recently declared result of first professional MBBS Examination by University of Health Sciences (UHS), Lahore, Pakistan. These were the first exams conducted under the new “Curriculum 2K23” introduced by UHS last year. According to the news headline, 5589 candidates of 44 medical colleges took the exam, out of which 4945 passed and 622 failed. Thus, the success rate was 88.83 percent.^1^

Congratulations are in order to the Vice Chancellor of UHS, Professor Ahsan Waheed Rathore, for the exceptional leadership demonstrated in the successful implementation of the newly launched modular integrated curriculum 2k23 for the MBBS professional examination for its first session of 2023-24. This landmark symbolizes a momentous achievement in medical education, not only across the 44 affiliated medical colleges of Punjab but also for the history of medical education in Pakistan where the largest serving public sector medical university has successfully launched the integrated curriculum for MBBS keeping with the standards of World Federation in Medical Education (WFME). The coherent planning and successful execution of a standardized examination process across the diversified students’ population in Punjab highlights the commitment of UHS Lahore towards safeguarding the quality and transparency of medical education system in the region with zero tolerance for slackness.

The integrated curriculum embodies a progressive step towards modernizing medical education in line with the contemporary healthcare demands.^2^ Though it had been implemented in a few medical schools in Pakistan for more than a decade but the initiative taken by the worthy Vice Chancellor to implement this new system of teaching, learning and assessment involving more than 6000 students and 2000 faculty members of courses related to the first professional examination across 44 medical schools has set an unprecedented example in Pakistan. The reason behind this claim is that for the first time in the history of Pakistan, any medical University has introduced the concepts of (1) *‘No internal Examiner’*, (2) ‘*100% observed OSPE (Objective structured practical examination)’*, (3) replacement of traditional viva with the *‘OSVE (Objective structured viva examination)’* and (4) ‘*entirely neutral venues for examinations’* for all public and private sector medical colleges across Punjab. Such initiatives are imperative for producing competent healthcare professionals equipped to address the evolving healthcare challenges of the 21st century.

Though it wasn’t a laid-back journey where UHS team had frequently encountered uproars, agitation and impediments laid or faced by many stakeholders but with the mutual and continuous collaboration, support and reassurance among all the stakeholders, this strategic implementation of the curriculum was made possible over the year 2023 while ensuring fairness and meritocracy in the evaluation of students’ performance.

As we commend the Vice Chancellor and the Medical Education Department of UHS led by Col. (Rtd) Dr. Khalid Rahim Khan and his entire team involved in this endeavor, let’s not forget to concede the challenges that lie ahead; maintaining the standards through evidence-based best practices and continuous quality improvement in medical education. Moving forward, UHS aims to build upon this success by incorporating feedback from stakeholders, identify and address any gaps or deficiencies in the curriculum, leveraging technological advancements, and adapting to emerging trends in healthcare and education for the upcoming batches. The wide geographical and administrative scope of UHS, coupled with the diverse nature of its students necessitates continuous monitoring, evaluation and improvement of teaching and learning methodologies. For this purpose, the Medical Education department along with the examination department has devised a stringent plan for continuous Block-based monitoring and evaluation of the taught courses, formative and continuous internal assessments, and progress shown by each medical college through a recently developed online portal system.

By fostering a culture of continuous improvement and collaboration, UHS with the support of its affiliated and constituent colleges, can further enhance its educational contributions by producing medical graduates who are not just competent but exemplary professionals, capable of competing with the best medical students on a global scale.
